# Cadherins in collective cell migration of mesenchymal cells

**DOI:** 10.1016/j.ceb.2012.08.002

**Published:** 2012-09-01

**Authors:** Eric Theveneau, Roberto Mayor

**Affiliations:** Department of Cell and Developmental Biology, University College London, UK

## Abstract

Immunity, embryogenesis and tissue repair rely heavily on cell migration. Cells can be seen migrating as individuals or large groups. In the latter case, collectiveness emerges via cell-cell interactions. In migratory epithelial cell sheets, classic Cadherins are critical to maintain tissue integrity, to promote coordination and establish cell polarity. However, recent evidence indicates that mesenchymal cells, migrating in streams such as neural crest or cancer cells, also exhibit collective migration. Here we will explore the idea that Cadherins play an essential role during collective migration of mesenchymal cells.

## Introduction

Collective cell migration, the coordinated migration of a cell population through cell-cell cooperation, is a recognized mode of migration during morphogenesis, wound healing and cancer metastasis [1–3]. Such collective behaviour was thought to be restricted to epithelial cells maintaining stable cell–cell adhesions, but recent data indicate that mesenchymal cells can also cooperate and undergo collective cell migration [4•,5•]. Mesenchymal cells are produced by Epithelial-Mesenchymal Transition (EMT). This complex process includes a cell–cell dissociation step during which stable cell contacts are downregulated [6]. In this review, we focus on the function of classic cadherins (type I and II) in collective movement. We start with a brief overview of the current knowledge of Cadherins' functions in epithelial tissues, including the dynamics of epithelial cell interactions and epithelial cell sheet migration. We then go on to propose a role and discuss possible mechanisms for these molecules in collective movement of mesenchymal cells.

## Cadherins in epithelial tissues

**Figure 1:**
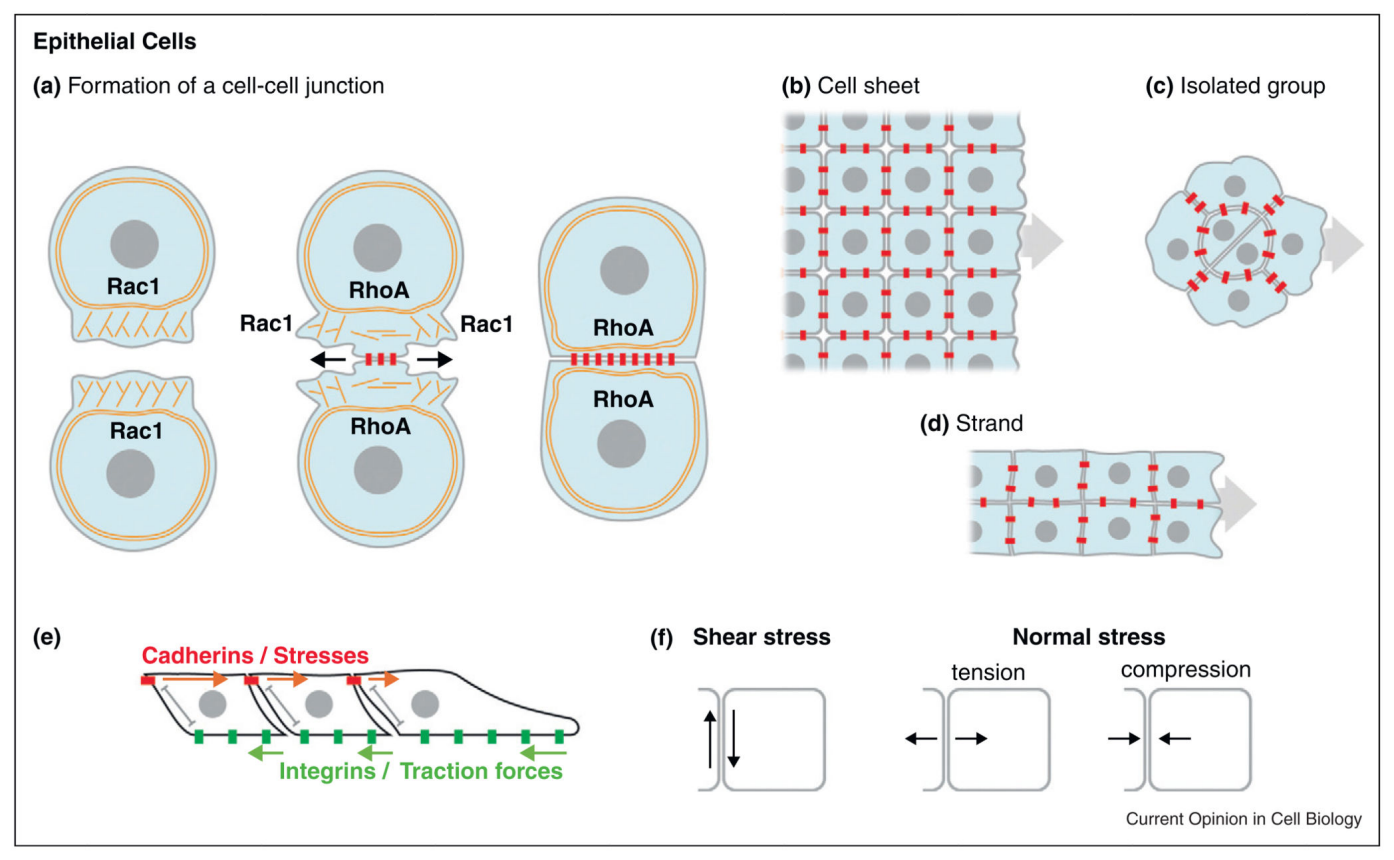
Cadherins in epithelial cells. **(a)** Interaction between two epithelial cells. Explorative protrusions driven by Rac1 activity promote the formation of an initial contact. At the site of contact RhoA controls the switch from branched actin to parallel bundles of actomyosin. The contact progressively expands into a stable Adherens Junction. **(b**–**d)** Different types of epithelial collective cell migration: cell sheet **(b)**, isolated groups **(c)** and strands **(d)**. **(e)** In such tissues, traction forces from integrin-mediated contacts with the extracellular matrix are transmitted as local stresses across the cell sheet via cadherin-based junctions. **(f)** Shear and normal stress are generated at the cell-cell contact. Cadherins are in red, integrins are in green. Actin cytoskeleton is shown as orange fibers.

Classic Cadherins are transmembrane proteins that engage in calcium-dependent homophilic bindings via their first extracellular domain [7]. Their interaction promotes the formation cell–cell junctions called Adherens Junctions (AJs) [8]. AJs contain Cadherins at only 10% of their maximum density and thus promote a relatively weak cell-cell adhesion compared with Desmosomes or Tight Junctions [8], although the binding affinity between these different molecules could also have an important role in determining the strength of cell-cell adhesion. New cell-cell adhesions are formed in a 3-step manner: initiation, expansion and stabilization (Figure 1a, [9]). Briefly, in the initiation phase, cells explore their local environment using protrusions, such as lamellipodia, to favour random encounter with nearby cells [10]. When membranes of two cells collide, cadherins present on their surface make homophilic contacts. Cadherin engagement induces a very transient peak of Racl activity directly followed by an increase of RhoA activity [11]. Consequently, the lamellipodial activity is inhibited at the nascent contact and progresses sideways. The wave of membrane activity on both sides promotes the formation of new adhesion sites by favouring membrane overlap. In the meantime, at the site of contact, branched actin is progressively converted into bundles of actomyosin parallel to the cell cortex [12••]. This polymerization of actin and actomyosin generated tension is the main driving force for the expansion of the cell-cell junction [10,13,14•]. The membrane activity and actin turnover progressively decrease as the region of contact grows larger. This helps to stabilize the connection between the cell adhesion complex (cadherin/catenins) to the cytoskeleton. In this context, activities of small GTPases must be extremely fine-tuned. For instance, Rac1 activity is essential for membrane exploration at nascent junctions, but maintaining Rac1 prevents maturation and eventually disrupts the junction. Similarly, Rho activity is essential for AJs expansion via contractile forces. However, premature contractility can destabilize young junctions unable to withstand the local forces, while excessive contractility disassembles mature ones [14•,^15^–^17^]. Thus, the series of events that follows within seconds of Cadherin engagement at nascent junctions determines if the junction will grow and mature or disassemble quickly. The molecular details underlying the fine-tuning of small GTPase activity during AJs formation remain elusive.

Cadherins attach to the cytoskeleton via their intracytoplasmic domain in two ways. The C-terminal part contains a β-catenin binding domain and β-catenin can then recruit α-catenin (reviewed in [13]). The role of α-catenin remains controversial since α-catenin does not seem to bind β-catenin and actin at the same time. However, it can recruit other actin-binding partners such as Vinculin [18] and Afadin [19]. In addition, it has been proposed that in regions where α-catenin concentration is high (i.e. at stable AJ), some α-catenin may detach from Cadherins and bind to actin as a dimer, where it competes with the Arp2/3 complex. This mechanism would prevent actin branching at the site of cadherin homophilic interactions and thus promote the formation of parallel actin bundles. Therefore a-catenin seems to have a dual role at the junction: linking cadherins to microfilaments, via its ability to recruit actin-binding proteins to the cytoplasmic tail of cadherins, and preventing actin branching by competing with Arp2/3 when released in the cytosol [8,20,21]. In addition to microfilaments, AJs can also interact with microtubules. The juxtamembrane domain of Cadherins contains a p120-catenin binding site. p120 can link Cadherins to microtubule plus-ends via dynein (a minus-end molecular motor) and to the minus-end via PLEKHA7 and Nezha (reviewed in [8]). AJs and the cytoskeleton are interdependent. Assembly, recycling and stabilization of Cadherin is controlled by its interaction with the cytoskeleton, but Cadherin engagement also controls cytoskeletal rearrangement (reviewed in [8,9]).

**Figure 2:**
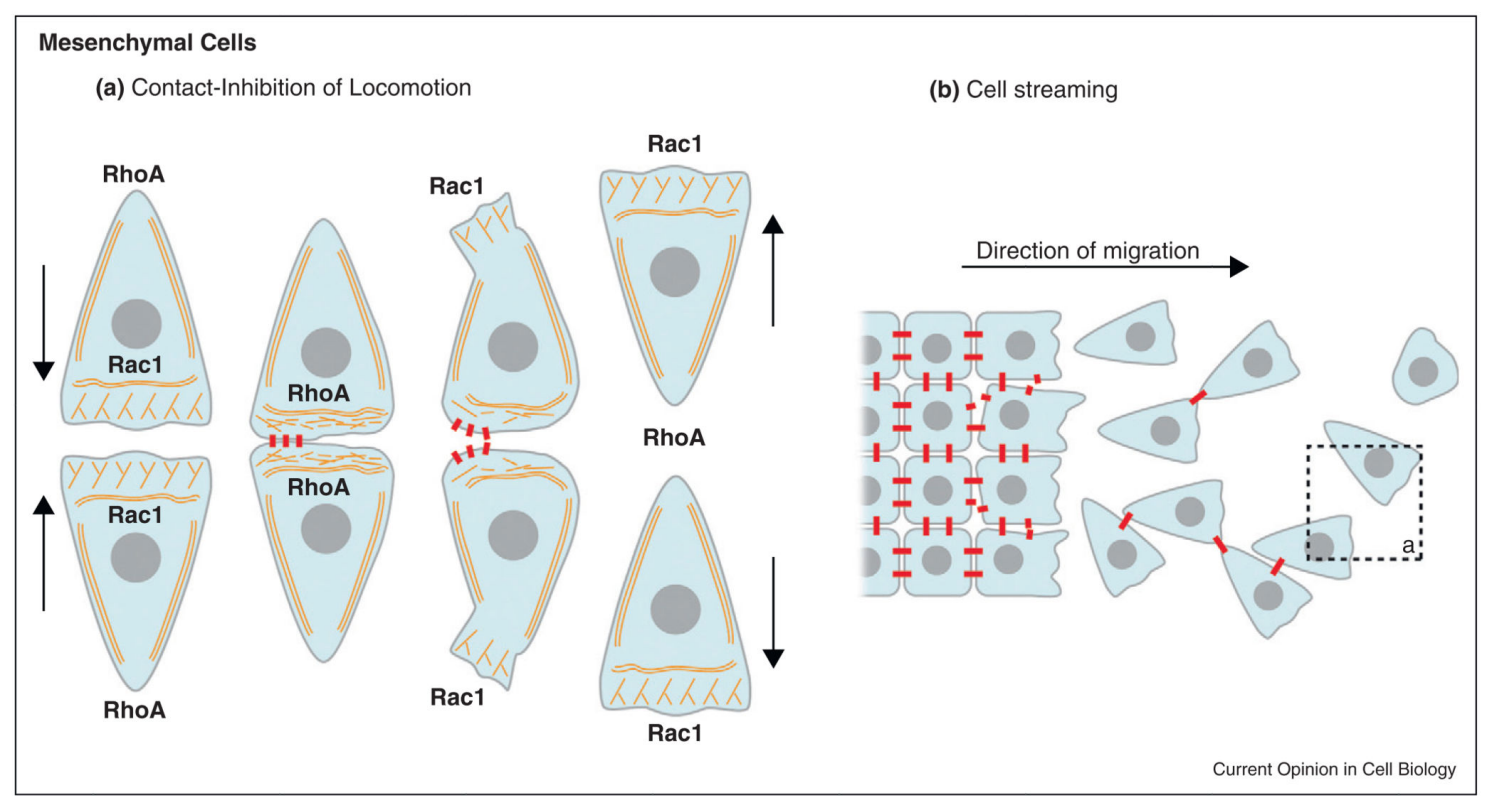
Cadherins in mesenchymal cells.**(a)** Interaction between mesenchymal cells leading to Contact-Inhibition of Locomotion (CIL). Cadherin-dependent contacts are transiently established between the colliding cells. The contact inhibits protrusive activity and is followed by a peak of RhoA activity that induces retraction of the cell body. Both cells repolarize in opposite directions and move away from each other. **(b)** An epithelial tissue undergoes EMT. Cells progressively lose their cell-cell adhesion and start migrating as a stream of individual cells and small groups of loosely associated cells. When two cells collide they exhibit CIL (dotted line, see “a”). Cells are show as blue shapes with a grey center representing the nucleus. Cadherins are in red. Actin cytoskeleton is shown as orange fibers.

**Figure 3:**
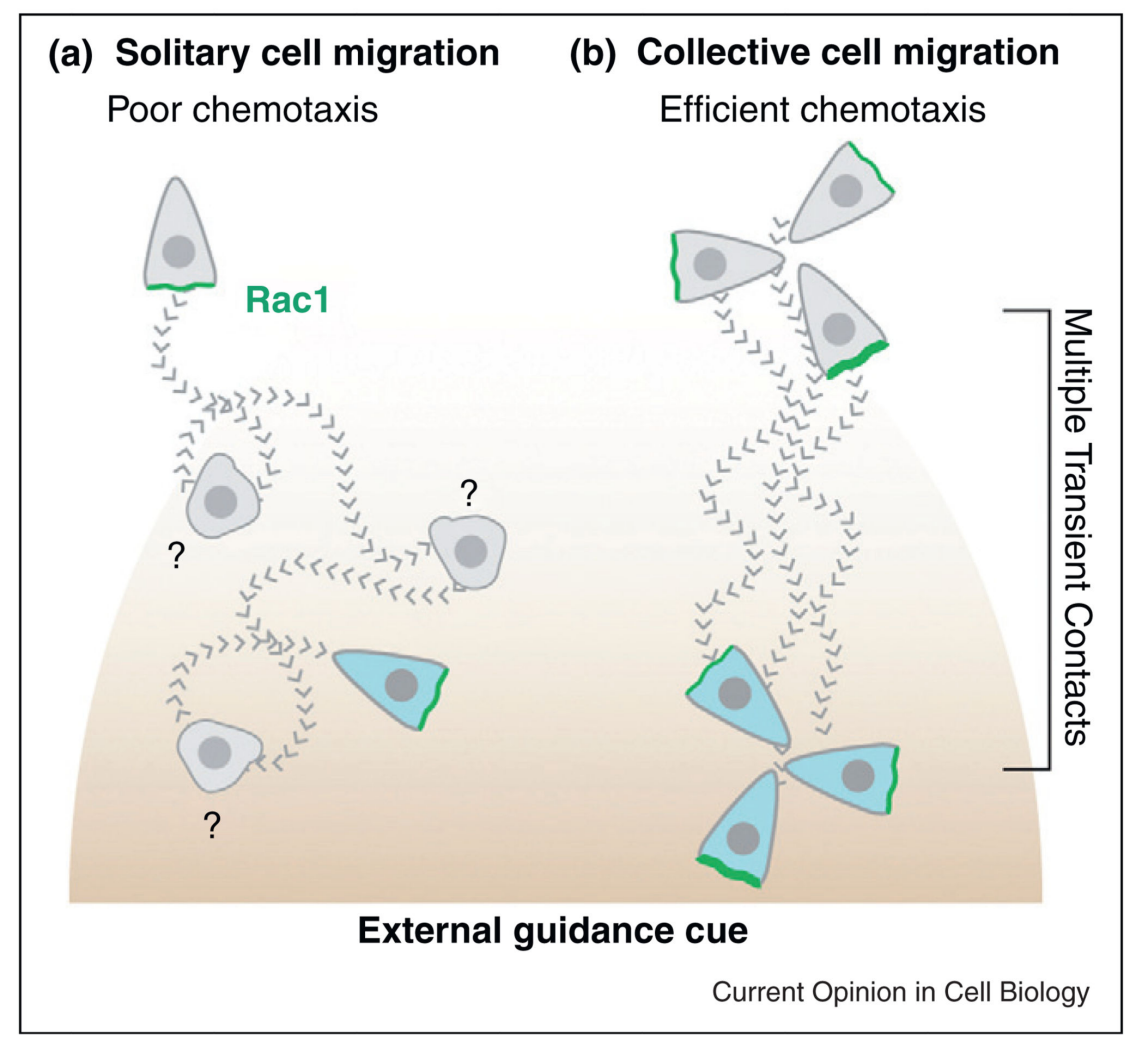
Collective migration enhances chemotaxis in Neural Crest cells.**(a)** Individual cells show poor chemotactic abilities when placed in a gradient. Weak transient protrusions form at random and the attractant is mostly inefficient at modulating them. **(b)** Cells at high cell density constantly collide with each other. Each collision strongly repolarizes the cells. The attractant positively biases the well-oriented protrusions very efficiently. That is sufficient to confer an overall directionality onto the cell population. Grey cells represent earlier time points. Migratory paths are shown as dotted lines. Question marks indicate phases of reorientation during which cell polarity is lost. Green represents Rac1.

Epithelial tissues can move as sheets, strands or isolated groups (Figure 1b–d, [2,3]) and Cadherins have been shown to play an important role in their coordinated migration. For instance, dynamics of blood vessel sprouting relies on VE-Cadherin [22,23], posterior Lateral Line Primordium of the Zebrafish express several Cadherins and loss of function experiments targeting these molecules impair migration [24–27], while some cancer cells undergo Cadherin-dependent migration [14•,28•,^29^–^31^]. Several studies on directional migration of expanding cell sheets in 2D-cultures highlighted the role of AJs in cell coordination [32•,33••]. Control epithelial cells exhibit highly directional movement while inhibition of E-Cadherin increases randomness. Interestingly, direct measurements of forces across the cell sheet showed that traction forces from integrin-matrix interactions lead to a build up of tension across the tissue (Figure 1e). Thereare multiple forces occurring at the cell-cell contact, such as shear and normal stress, which are parallel and orthogonal to the cell-cell interface, respectively (Figure 1f). Cells align in the direction of the maximum normal stress and minimal shear stress, being these stresses transmitted through AJs [33••,^34^–^36^]. In the case of a cell sheet attempting to close a wound, such cell alignment mechanism based on transmission of stresses allows cell polarity to be generated in the direction of the space to be filled, without gaps forming within the population itself. Tissue integrity, via maintenance of AJs, is used as a means of converting an anisotropic situation (appearance of a free edge owing to a wound) into a global reorganization of the tissue via progressive cell alignment along the direction of transmitted stress.

In the Drosophila egg chamber, a small cluster of cells, called the Border Cells, travels between Nurse Cells from one end of the chamber to the oocyte [37]. Border cells express E-Cadherin between them and this is essential for these cells to polarize. However, the local environment through which they migrate does not contain extracellular matrix and E-Cadherin is also used to establish contact with the surrounding Nurse Cells [38•,^39^,^40^]. Remarkably, these E-Cadherin junctions between Border cells and Nurse cells are compatible with the formation of cell protrusions, while AJs between Border cells are not and restrict protrusive activity outward. This suggests that two types E-cadherin engagements, with two different outcomes, co-exist in Border cells. This highlights the importance of deciphering the actual molecular composition of specific Cadherin-based junctions to understand how they might lead to cell protrusions, stable AJs or transient contacts.

In conclusion, the use of Cadherin-based junctions during collective cell migration of epithelial cell population is extremely diverse. Cadherins can be used to transmit signals via local stress and tension, to polarize cells by restricting formation of cell protrusions away from the contact and to promote interaction with surrounding tissues if needed.

## Cadherin-based junctions in collective cell migration of mesenchymal cells

Mesenchymal cells are produced by an EMT [6]. They have lost stable cell-cell junctions but usually keep expressing various Cadherins that are present at their surface. However, EMT is not an all-or-nothing event, as there is a continuous gradation from a complete EMT, such as in melanocytes, to partial EMT, such as Xenopus mesoderm (gradation of EMT is reviewed in [41]). Cadherin-based contacts are involved in the migration of many different mesenchymal-like cell types such as myofibroblasts [42], neurons and glial cells [43–45]. As examples of mesenchymal cells, we will focus on mesodermal and Neural Crest (NC) cells [1,46,47].

### Collective migration of mesodermal cells

Mesoderm is a germ layer formed during early embryo development that moves from an external to a more internal position within the embryo during gastrulation. Although mesodermal cells are a typical example of mesenchymal cells, not always they undergo a complete EMT, such as Xenopus mesoderm, which migrate as a pseudo-epithelial cell sheet (a motile group without complete cell-cell dissociation) [48•]. The idea of stress-dependent polarity discussed above for typical epithelia cells has also been explored in Xenopus mesodermal cells. These cells are connected through C-Cadherin dependent junctions. Interestingly, C-Cadherin engagement in absence of stress does not have an effect on cell polarity. However, when local stress is applied on C-Cadherin, cells repolarize away from the region of stress by forming a protrusion in the opposite direction [48•]. These observations are in accordance with cross-talks between Cadherin-based junctions and cell-matrix interactions reported by several groups [49–53].

Migration of the mesoderm in zebrafish has been widely studied and it relies on E-Cadherin [54]. In this system, cells migrate collectively but cells that are experimentally isolated can migrate as efficiently as groups. However, groups without E-Cadherin fail to successfully undergo directional migration [55••] suggesting that collectiveness mediated by AJs is only required when cells are at high cell density. In this case, a high cell density is thought to affect the distribution or availability of guidance cues. For instance, leader cells may degrade or shield signals from followers. Therefore, connections via AJs are required to couple cells in order to reduce variations across the population.

### Collective migration of neural crest cells

Neural crest (NC) is an embryonic cell population that undergoes delamination after EMT [1,56,57]. NC cells have been shown to exhibit localized N-Cadherin-based contacts and gap junctions, which are both important for efficient migration [5•,^58^–^65^]. There is evidence that NC cells from Xenopus, zebrafish and chick exhibit Contact-Inhibition of Locomotion [66,67,68••] (CIL, Figure 2a) and migrate as a loose but dense collective (Figure 2b). CIL is the process by which a cell ceases moving after being contacted by another cell [68••,^69^–^71^] and is often described as having two phases: a collapse of the cell protrusions upon contact that leads to a transient arrest of migration and a repolarization in the opposite direction with cells eventually moving away from each other. In a mesenchymal cell population at high cell density or in cells that retain a pseudoepithelial phenotype, CIL prevents the formation of cell protrusions in between neighbours. Thus, most of the protrusive activity is directed towards the free space [5•,68••,^72^].

When two NC cells collide, RhoA activity increases at the contact [68••] while that of Rac1 decreases [5•]. These events depend on N-Cadherin and Wnt/PCP signalling [5•,^68^••,^73^]. The lamellipodium collapses but instead of propagating laterally to expand the contact area, as observed during epithelial cell-cell interaction, a new lamellipodium is formed on the opposite side of the cell (Figure 2a). In addition, RhoA activity does not promote the reorganization of the actin cytoskeleton parallel to the region of contact and the cell-cell junctions are not reinforced. Instead, cells contract their cell body to move away from each other in a RhoA/Rock-dependent mechanism [68••]. Why this local activation of RhoA upon Cadherin binding leads to two opposite behaviours in epithelial versus mesenchymal cells remains unknown. It has been shown that actomyosin activity needs to be maintained at low levels to allow long-lasting cell-cell junctions while high levels of RhoA activity promote actin bundle formation at the basal side of the cells and lead to retraction of the cell rear and junction disassembly [16,17,74,75••]. However, quantification of absolute levels of RhoA has remained beyond reach. Importantly, despite lacking stable cell–cell contacts, NC cells cooperate and undergo collective migration [5•]. This is clear when cells are exposed to an external gradient of chemotactic cue. Isolated cells chemotax poorly (Figure 3a) while individual cells cultured at high cell density respond efficiently (Figure 3b, [5•]). A similar cooperation has been observed in Xenopus mesodermal cells [76]. How cooperation is mediated remains elusive. One possibility is that the transient contacts not only polarize the cells but also control the local distribution of surface receptors that are important for chemotaxis. It is also unclear if these local N-Cadherin contacts lead to the formation of proper, even though transient, AJs containing the molecular effectors essential for cytoskeleton remodelling.

**Figure 4:**
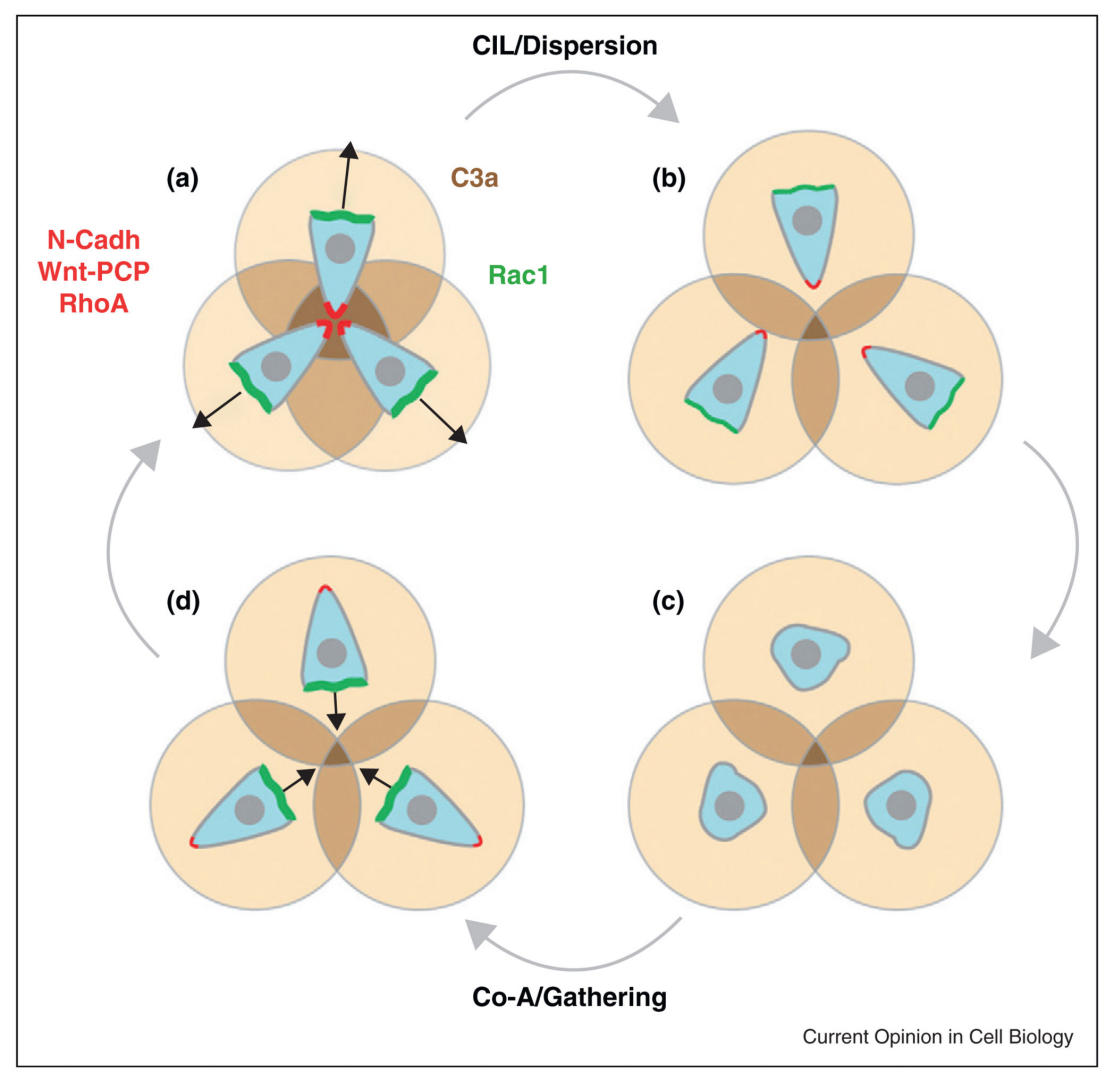
Contact-Inhibition and Co-Attraction balance each other out to maintain high cell density in migratory Neural Crest cells.**(a)** NC cells are polarized according to their cell–cell contact owing to CIL, with protrusions oriented outward. **(b)** CIL leads to cell dispersion. **(c)** In the absence of contact cells quickly lose their polarity. **(d)** Each cell repolarizes according to the local concentration of C3a (shades of brown), owing to co-attraction (CoA). This promotes gathering. Cells moving towards each other eventually collide and repolarize again owing to contact-inhibition.

Because CIL promotes protrusion collapse and repolarization, mesenchymal cells that exhibit CIL quickly disperse. Therefore, some backup system must prevent extensive dispersion in order to maintain a critical cell density allowing collectiveness to emerge. *In vivo*, NC cells are surrounded by local inhibitory signals that restrict their migration into specific territories [1,46,47]. In addition, each NC cell expresses a chemoattractant and its cognate receptor: complement factor C3a and C3aR, respectively (Figure 4, [4]). C3a is a complement factor with well characterized chemoattractant activity in the immune system [77]. When a NC cell leaves the main group, it moves back towards the region of high cell density by following the local gradient of C3a produced by each NC cell, in a process called co-attraction (Figure 4). This is possible because C3a/C3aR signalling activates Rac1, which promotes the formation of a new protrusion [4]. When cells rejoin the group, a new N-Cadherin-dependent contact is established that leads to CIL and dispersion (Figure 4). The presence of C3a and its receptor has been shown for NC from Xenopus [4], mouse (Lambris and Mayor, unpublished) and chick (Bronner and Mayor, unpublished), and for mesoderm of Xenopus embryos [78]. Interestingly, cerebellar granule neurons have been shown to use tip-like N-Cadherin-based contacts to migrate as chains [79•] and some tumours express autocrine chemotactic factors and Cadherins that would allow a cycle of CIL and mutual attraction to emerge [80–82]. Furthermore, it has been recently shown that during the migration of zebrafish lateral line primordium, some isolated lateral line cells are attracted by chemoattractants produced by the clustered primordium cells [83], in a process similar to the coattraction described for NC cells.

Xenopus NC cells also express Cadherin-11 [84]. Intriguingly, Cadherin-11 is found at the leading edge of the cells where it seems to regulate small GTPases and favour filopodia and lamellipodia formation [85]. Cadherin-11 is cleaved by Adam13 and is therefore present as a full length protein, a transmembrane portion and as a soluble extracellular fragment [86]. Specific functions of these different forms are yet to be determined but these data suggest that Cadherin processing may play a role in the regulation of cell–cell and cell-matrix interactions.

## Perspectives

Studies on coordination through transmission of forces in epithelial and pseudoepithelial cell sheets have provided an explanation for how AJs may transmit and integrate changes in cell polarity allowing a complete reorganization at the tissue level. How cell cooperation emerges in mesenchymal cells is unclear. Are actual AJs transiently formed upon cell–cell collisions during CIL? Are Cadherins linked to the cytoskeleton during transient contact? Are these transient cell–cell interactions sufficient to promote transmission of forces? Are Cadherins signalling or just bringing membranes together to favour activation of other pathways such as non-canonical Wnt/PCP or to promote formation of Gap Junctions? These are some of the questions that will have to be addressed in order to better define what cooperation in mesenchymal cells actually means.
